# Macrophage Heterogeneity and Plasticity: Impact of Macrophage Biomarkers on Atherosclerosis

**DOI:** 10.1155/2015/851252

**Published:** 2015-09-27

**Authors:** Joselyn Rojas, Juan Salazar, María Sofía Martínez, Jim Palmar, Jordan Bautista, Mervin Chávez-Castillo, Alexis Gómez, Valmore Bermúdez

**Affiliations:** ^1^Endocrine and Metabolic Diseases Research Center, School of Medicine, University of Zulia, Maracaibo 4004, Venezuela; ^2^Endocrinology Department, Maracaibo University Hospital, Maracaibo 4004, Venezuela

## Abstract

Cardiovascular disease (CVD) is a global epidemic, currently representing the worldwide leading cause of morbidity and mortality. Atherosclerosis is the fundamental pathophysiologic component of CVD, where the immune system plays an essential role. Monocytes and macrophages are key mediators in this aspect: due to their heterogeneity and plasticity, these cells may act as either pro- or anti-inflammatory mediators. Indeed, monocytes may develop heterogeneous functional phenotypes depending on the predominating pro- or anti-inflammatory microenvironment within the lesion, resulting in classic, intermediate, and non-classic monocytes, each with strikingly differing features. Similarly, macrophages may also adopt heterogeneous profiles being mainly M1 and M2, the former showing a proinflammatory profile while the latter demonstrates anti-inflammatory traits; they are further subdivided in several subtypes with more specialized functions. Furthermore, macrophages may display plasticity by dynamically shifting between phenotypes in response to specific signals. Each of these distinct cell profiles is associated with diverse biomarkers which may be exploited for therapeutic intervention, including IL-10, IL-13, PPAR-*γ*, LXR, NLRP3 inflammasomes, and microRNAs. Direct modulation of the molecular pathways concerning these potential macrophage-related targets represents a promising field for new therapeutic alternatives in atherosclerosis and CVD.

## 1. Introduction

Cardiovascular disease (CVD) is currently recognized as the leading cause of morbidity and mortality in the adult population worldwide, with an estimated projection of 23.3 million yearly deceases attributable to these disorders by the year 2030 [1, 2]. Data from our country reflect this global trend, with CVD being responsible for 20.9% of total mortality in Venezuela [3]. This surge in cardiovascular morbidity and mortality has been associated with increased prevalence of cardiometabolic risk factors and ineffective preventive strategies in primary care and public health management [4]. The severe epidemiologic and socioeconomic implications of CVD have propelled increased scientific interest into its pathophysiology, particularly atherosclerosis. Although multiple risk factors intervene in this process, recent findings suggest immunologic phenomena may play key roles in its evolution [5].

As part of the innate immune system, monocytes actively participate in the development of atherosclerosis by infiltrating the subendothelium in vascular walls. This process is facilitated by endothelial dysfunction, which is in turn propitiated by various cardiovascular risk factors [6]. Dysfunctional endothelium releases proinflammatory cytokines, such as Interleukin-6 (IL-6) and tumor necrosis factor-*α* (TNF-*α*), and growth factors and expresses multiple cell adhesion molecules which allow recruitment and adhesion of monocytes to vessel walls, promoting their differentiation to activated macrophages capable of phagocytizing oxidized low-density lipoprotein (oxLDL) present in the subendothelium [7, 8].

This cellular differentiation may yield several distinct subtypes of macrophages in response to numerous microenvironmental signals modulating genomic expression in these cells [9]. This variable outcome has originated the concepts of monocyte and macrophage “heterogeneity” and “plasticity,” alluding to their ability to specialize into and switch between distinct functional phenotypes in the presence of different environmental cues, including cytokines, microbial products, oxLDL, among others [10]. These properties appear promising regarding their potential as therapeutic targets in CVD, as they may allow attenuation of the proinflammatory milieu in atherosclerotic lesions [11]. This review discusses the molecular basis of monocyte and macrophage heterogeneity and plasticity and their implications for future clinical intervention.

## 2. Monocyte Heterogeneity

**Figure 1:**
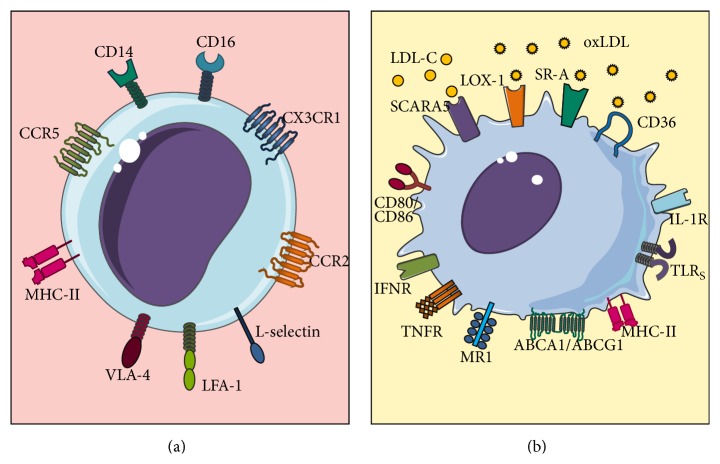
Features of monocytes and macrophages. (a) Classic monocyte. (b) Classic macrophage. Both classic monocytes and classic macrophages play essential roles in innate immune responses, expressing an ample variety of receptors that modulate their activation.

Monocytes stem from a myeloid precursor in the bone marrow, from where they are released into circulation, showing a relatively short half-life (1–3 days in humans) and a strong ability to adhere to the vascular endothelium and penetrate tissues in response to endothelial and subendothelial signals [12]. Following extravasation, monocytes localize to the subendothelium, where they may differentiate into distinct subtypes of macrophages, supported by several growth factors, mainly Macrophage Colony-Stimulating Factor (M-CSF) [13] and Granulocyte-Macrophage Colony-Stimulating Factor (GM-CSF) [14], Figure 1.

**Figure 2:**
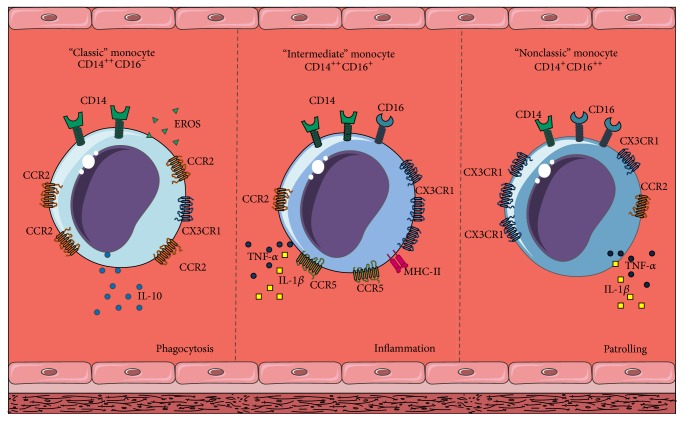
Monocyte heterogeneity. According to differential expression of specific cell surface markers and receptors, monocytes may be classified into three distinct subpopulations: “classic” monocytes [CD14^++^CD16^−^], “intermediate” monocytes [CD14^++^CD16^+^], and “nonclassic” monocytes [CD14^+^CD16^++^].

Prior to differentiation, monocytes may show a range of distinct phenotypes according to the differential expression of specific cell surface markers [32], which are subject to environmental modulation. These markers include the Lipopolysaccharide (LPS) receptor antigen (CD14), present in almost all monocytes, and the IgG Fc*γ* receptor (CD16), expressed only in select groups of these cells [32]. Monocytes may be categorized in 3 subpopulations according to the presence of these components: “classic” monocytes [CD14^++^CD16^−^], “intermediate” monocytes [CD14^++^CD16^+^], and “nonclassic” monocytes [CD14^+^CD16^++^] [33, 34]. Each of these types has been reported to exhibit significant differences regarding expression of cell adhesion molecules and chemokine receptors, both of which are pivotal for adhesion and recruitment to the dysfunctional endothelium [35], Figure 2.

Classic monocytes [CD14^++^CD16^−^] constitute 80–95% of total circulating monocytes and primarily act as phagocytes, boasting strong peroxidase activity and predominantly releasing Interleukin-10 (IL-10) in response to LPS [33, 34]. They also express high levels of MCP-1 receptor (CCR2) and L-Selectin (CD26L), alongside low levels of CX3C-1 chemokine receptor (CX3CR1), allowing quick recruitment to inflammatory signal-generating sites [34, 36]. This cellular subset has been identified as the main monocyte subtype involved in the inflammatory process at the atheromatous plaque, fundamentally due to their increased expression of CCR2 [37]. Furthermore, CCR2 in these cells may be a potential therapeutic target for modulation of their recruitment. In this regard, silencing of CCR2 in Ly-6C^hi^ monocytes in murine models, which are equivalent to CD14^++^CD16^−^ monocytes in humans, has been linked to attenuation of the inflammatory response associated with atherosclerosis and myocardial infarction [38].

On the other hand, intermediate monocytes [CD14^++^CD16^+^] represent 2–10% of total circulating monocytes, show minimal peroxidase activity, and secrete large quantities of Interleukin-1*β* (IL-1*β*) and TNF-*α* in response to LPS; thus, their role is preeminently proinflammatory, intensely expressing CXCR-1 and moderate amounts of CCR2 [33, 34]. Intermediate monocytes also express high levels of C-C chemokine receptor type 5 (CCR5), whose main ligand is CCL5, an important chemotactic molecule for T cells, allowing this subpopulation of monocytes to participate in activation of T cells and amplification of local inflammatory activity [33, 34].

Finally, nonclassic monocytes [CD14^+^CD16^++^] comprise only 2–8% of total circulating monocytes and are considered “patrolling” or “surveillance” cells because they express low levels of CCR2 and high levels of CX3CR1, resulting in great endothelial affinity with a stunted response to chemotaxis [32, 39].

## 3. Macrophage Heterogeneity

Macrophages play a crucial role in immune responses, by actively participating in a myriad of biological processes, such as resolution of infections and repairing of injured tissues, as prompted by numerous signals, which include microbial molecules and proinflammatory cytokines [40]. Following differentiation from monocytes, macrophages may adopt various functional phenotypes as directed by diverse stimuli [41], a process that is species-specific and very finely regulated [42].

Macrophages adopt the M1 phenotype following binding of Interferon-*γ* (IFN-*γ*) to its extracellular heterodimeric receptors IFNGR-1 and IFNGR-2, which initiates JAK1- and JAK2-mediated signaling cascades, culminating in activation of STAT1, which in turn activates the transcriptome for the M1 profile [43]. These cells exhibit proinflammatory and phagocytic traits, expressing abundant membrane scavenger receptors which permit endocytosis of modified lipoproteins such as oxLDL [44].

**Figure 3:**
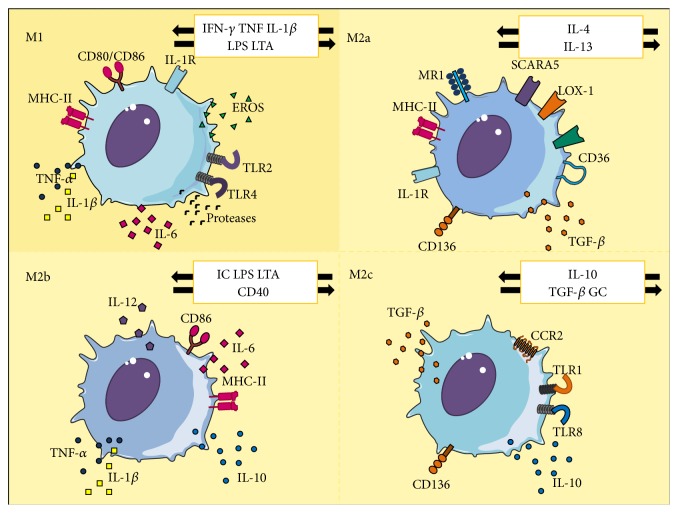
Macrophage heterogeneity. Monocyte activation and differentiation towards distinct macrophage subpopulations (M1 or M2) hinges on the predominant pro- or anti-inflammatory microenvironment within the lesion. M1 macrophages result from classic monocyte activation in response to proinflammatory stimuli, such as IFN-*γ*, TNF, IL-1*β*, LPS, and LTA. On the other hand, M2 macrophages are subdivided into M2a, activated by IL-4 and IL-13; M2b, activated by IC, LPS, LTA, and CD40; and M2c, activated by IL-10, TGF-*β*, and GC. IFN-*γ*: Interferon-*γ*, TNF: Tumoral Necrosis Factor, IL-1*β*: Interleukin-1*β*, LPS: Lipopolysaccharide, LTA: lipoteichoic acid, IL-6: Interleukin-6, IL-4: Interleukin-4, IL-13: Interleukin-13, TGF-*β*: Transforming Growth Factor-*β*, IC: immune complexes, and GC: glucocorticoids.

The concept of macrophage “*heterogeneity*” was introduced after observations of monocytes undergoing macrophagic differentiation through an alternate route, towards a cellular subpopulation with different characteristics, termed M2 macrophages [40, 45]. Moreover, this alternative pathway may originate at least three well-defined macrophage subtypes: M2a, M2b, and M2c [46, 47], Figure 3. IL-4, released by Th2 lymphocytes, eosinophils, basophils, and macrophages, appears to be the principal messenger triggering the switch M2 [48]. After binding to its receptor (IL-4R), this cytokine starts JAK1- and JAK3-mediated intracellular cascade that finalizes in activation of STAT6, which then drives M2 differentiation [48]. IL-10 may also favor this switch through activation of JAK1-STAT3 [49], and glucocorticoids may directly promote transcription of M2 genes and silencing of M1-related genes, as part of their robust anti-inflammatory capabilities [50].

Each kind of M2 macrophage displays distinct biologic profiles. M2a are strongly anti-inflammatory, inhibiting release of INF-*γ*, IL-1, IL-6, IL-10, GM-CSF, and TNF-*α*, have poor phagocytic capacity, and contribute to deposition of extracellular matrix by potentiating synthesis of polyamines, collagen, and Transforming Growth Factor-*β* (TGF-*β*); thus, M2a may be described as “tissue-repairing” macrophages [51]. On the other hand, M2b and M2c, known as “regulator” macrophages, can limit tisular damage caused by prolonged activation of M1 macrophages by modulating numerous aspects of chronic inflammation [52]. However, they have been shown not to actively participate in tissue repairing, as they are unable to synthesize extracellular matrix, and express different chemokine receptors, in contrast with M2a macrophages [53].

On the other hand, transition to M2b may be triggered by exposure to immune complexes, TLR ligands, LPS, lipoteichoic acid, and other intermediaries. These cells boast remarkable IL-10 secretion associated with FoxO1 expression [54] and very low IL-12 release, along with moderate secretion of TNF-*α*, IL-6, and IL-1 [55]. Lastly, M2c macrophages, whose activation may be driven by IL-10, TGF-*β*, or glucocorticoids, are also capable of abundant IL-10 release but are also able to inhibit production of proinflammatory cytokines, Nitric Oxide (NO), and Reactive Oxygen Species (ROS), outlining an important role in limiting progression of inflammation [56].

## 4. From Endothelial Dysfunction to Atherosclerosis: A Quick Revision

Atherosclerosis is a multifactorial, chronic, and progressive inflammatory process which develops from endothelial dysfunction [57]. The endothelium is a metabolically active organ, capable of producing a wide array of vasoactive messengers, notably NO, Prostacyclin I_2_ (PGI_2_), Endothelins (ET-1, ET-2, and ET-3), Thromboxane A_2_ (TXA_2_), and angiotensin II [58–60]. These mediators perform several functions, with some of them antagonistic in nature, in order to maintain vascular homeostasis in response to specific stimuli. The loss of this equilibrium, that is,* endothelial dysfunction,* sets the stage for the earliest phases of atherosclerosis [61]. This alteration is propitiated by various cardiovascular risk factors, such as dyslipidemia [62], uncontrolled Diabetes Mellitus (DM) [63], hypertension [64], and smoking [65]. These entities induce production of ROS, intensifying peroxynitrite-dependent oxidative stress and diminishing NO bioavailability by uncoupling the endothelial Nitric Oxide Synthase (eNOS) activity and transformation to peroxynitrite [66]. Other reactive species, including hydrogen peroxide, superoxide anions, and hypochlorous acid, also participate in this scenario, particularly by disrupting mitochondrial functionality [67].

The natural history of atherosclerotic disease may be studied in 6 continuous stages, from the formation of the lipid core to vascular lumen obliteration [68]. Elevated levels of low-density lipoprotein-cholesterol (LDL-C) appear to be essential mediators in Stage I, directly participating in the organization of the lipid core and promoting polarization of circulating monocytes towards proinflammatory phenotypes (CD68^+^CCR2^+^) [69, 70]. LDL-C molecules are especially susceptible to oxidation within the plaque, owing to their subendothelial localization. This is facilitated by a vascular lumen/subendothelium gradient which allows passage of LDL-C to the latter, with anchorage to heparan-sulfate [71], and by marked structural instability of LDL-C due to high concentration of polyunsaturated fatty acids (PUFA) [71]. Nevertheless, native LDL-C is recognized by Class B scavenger receptors, which play a limited role in macrophage differentiation [72].

In Stage II [68], PUFA in LDL-C are rapidly peroxidized, producing lipid hydroperoxides which subsequently degenerate, yielding a complex mix of byproducts, including malondialdehyde, 4-hydroxynonenal, and hexanal [73, 74]. These substances can interact with amino groups from Apoprotein B100 in LDL-C, modifying its structure via formation of Schiff bases (nonoxidative modification) between positively charged amino groups and aldehyde groups, producing modified LDL-C (mLDL-C). These molecules are highly antigenic and can be easily recognized by scavenger receptors for subsequent endocytosis and formation of foam cells [75].

Scavenger receptors are found in a broad array of cells, remarkably in macrophages, monocytes, platelets, endothelial cells, smooth muscle cells, and epithelial cells [76], mediating internalization of mLDL and other polyanionic ligands, such as oxLDL, acetylated LDL, Gram-positive and Gram-negative bacteria, apoptotic cells, and advanced glycation end products [77, 78]. Activation of these receptors is a key immunologic event in the development of atherosclerosis, as it promotes chemotaxis in monocytes and neutrophils, differentiation of macrophages, and formation of foam cells [79]. These changes in macrophage functionality remain for extended periods of time due to epigenetic histone modifications [80].

In this stage, oxLDL are particularly potent mediators within the subendothelium, given their strong antigenicity and their ability to induce expression of genes involved in the inflammatory response in endothelial cells and macrophages. For instance, they are able to increase endothelial permeability via multiple mechanisms, such as cytoskeletal disorganization through inhibition of myosin light-chain phosphatase, with activation of Rho kinase [81], and promotion of heparanase secretion with heparan-sulfate degradation in the extracellular matrix, facilitating extravasation of immune cells to the intimal space of dysfunctional endothelium [82].

Nevertheless, one of the most notable roles of oxLDL is the induction of Nuclear Factor-*κ*B (NF-*κ*B) [83–86], a key mediator in the perpetuation of the proinflammatory environment in the plaque [87], a crucial characteristic of Stage III [68]. Activation of NF-*κ*B leads to expression of several cellular adhesion molecules like P-Selectin, Vascular Cell Adhesion Molecule-1 (VCAM-1), and Intercellular Adhesion Molecule-1 (ICAM-1), indispensable for the fixation of immune cells to the endothelium during chemotaxis [88, 89]. Furthermore, Monocyte Chemoattractant Protein-1 (MCP-1), a 76-amino acid peptide of the C-C chemokine family [90], is also induced in endothelial cells and vascular smooth muscle cells (VSMC) when stimulated by oxLDL, angiotensin II, C-Reactive Protein, and TNF-*α* [91]. MCP-1 is considered the principal chemotactic intermediary involved in regulation of migration and infiltration of monocytes, T cells, and NK cells to the subendothelium in the earlier stages of atherogenesis [72]. During Stage IV [68], chemokines induce VSMC proliferation and inhibit NO synthesis, actively contributing to the evolution and progression of atherosclerotic lesions [91]. Finally, Stages V and VI convey the formation of foam cells, derived not only from macrophages but also from VSMC and endothelial cells, calcification of the lipid core, organization of the fibrous cap, and development of plaque vulnerability, which ends in plaque rupture and exposure of the matrix, activation of platelets, and acute formation of thrombi [68].

Although formation of macrophage-derived foam cells requires activity from several scavenger receptors, CD36 from Class B appears to be the main mediator [44, 72, 77, 79, 92]. The CD36 intracellular signaling cascade involves phosphorylation of syn (an src/yes-related human gene) [93], fyn, and lyn (*src* kinase) [94, 95], following activation of JNK1 and JNK2. This process culminates in oxLDL uptake via Vav, a guanine nucleotide exchange factor which acts as a scaffolding protein, modulating dynamin-dependent cytoskeletal processes [96]. Platelet-activating factor receptor [97] and Toll-like receptors (TLR) 4 and 6 [98] also appear to be required to be present in the same lipid raft as CD36 for oxLDL internalization. Activation of CD36 also triggers activation of inflammasomes and release of IL-1*β*, loss of cell polarity, stunting of cell movement, inhibition of autophagy, and, finally, cell death [99]. Other factors appear to intervene in formation of macrophage-derived foam cells, including Bone-Morphogenetic Protein (BMP) 4 via Smad1/5/8 signaling [100], thymic stromal lymphopoietin [101], Visfatin [102], and TRAIL (Tumor Necrosis Factor-related apoptosis-inducing ligand) [103] through induction of CD36 and/or scavenger receptor Class A (SR-A).

Development of foam cells derived from VSMC is another paramount aspect of advanced atherosclerotic lesions, due to their involvement in intimal thickening and stability of the fibrous cap [68, 104]. Several VSMC phenotypes have been described, each with distinct responses to lipoproteins in* in vitro* models [105, 106]. Immune cues, such as activation of TLR4, can induce formation of foam cells from VSMC via activation of NF-*κ*B and induction of Acyl-coenzyme A:cholesterol acyltransferase 1 (ACAT1) [107]. Furthermore, molecules such as ezetimibe and TRPV1 have been shown to impede formation of VSMC-derived foam cells by preventing cholesterol accumulation [108] and promoting autophagy [109], respectively, highlighting the importance of these processes in this context. Recent research has also shown these cells carry cholesterol loads larger than previously considered, possibly linked to their relatively lower capacity for cholesterol release via ABCA1 and their ability to transdifferentiate towards a macrophage-like phenotype in advanced atherosclerosis [110, 111]. Finally, endothelium-derived foam cells display unique characteristics, such as expression of VCAM-1, increased intracellular calcium movement, and high levels of heat shock proteins [112].

## 5. Macrophage Plasticity and Atherosclerosis: Echoing Dr. Jekyll and Mr. Hyde

In addition to the processes and macrophage subtypes described previously, macrophages may shift between these phenotypes in response to certain signals within their dynamic local microenvironment [113], a property known as “*plasticity.*” This mechanism allows adaptation to a myriad of possible changes in dysfunctional endothelium or damaged tissue, including acute oscillations in levels of lipids, ROS, proinflammatory cytokines, microbial products, or oxLDL [114]. Indeed, within the atheromatous plaque, microenvironmental cues define the predominating local macrophage phenotype [115]: M1 cells tend to accumulate in rupture-prone areas of the plaque, in contrast with M2 cells, which are principally found in stable regions of the atherosclerotic plaque, along with high IL-4 and IL-13 levels, away from the lipid nucleus [116], and are considered highly resistant to degeneration into foam cells [117]. Neither phenotype is particularly predominant in the fibrous layer of the atherosclerotic lesion [118].

As commented beforehand, the main command for M1 switching is IFN-*γ* signaling [43, 44], with participation of other intermediaries, including Interferon regulatory factors (IRF). In this respect, IRF5 has been demonstrated to propel polarization towards the M1 phenotype and induce release of IL-12 and IL-23, propelling a powerful T helper Th1-Th17 response [119]. Likewise, Notch signaling has been linked to increased M1 gene expression via IRF8 [120]. Interestingly, several microorganisms have been observed to trigger various intracellular pathways associated with M1 polarization, such as* Listeria monocytogenes*,* Chlamydia* ssp., and* Salmonella typhimurium* [121]. In fact,* C. pneumoniae* [122, 123],* Helicobacter pylori* [124], and* Cytomegalovirus* [125] have been identified in the atherosclerotic lesions, where they may promote polarization of the local macrophages towards the M1 phenotype and upregulate M1-related genes like TNF-*α*, IL-6, IL-1*β*, CCL2, CCL5, and CXCL8 [126].

Obesity and various obesity-associated disorders, such as DM and metabolic syndrome, have been linked with imbalanced M1/M2 ratios [127], propitiated by the chronic, subclinical, and proinflammatory state promoted by adiposopathy and insulin resistance [128] and chronic insulitis [129]. Moreover, increased levels of CD68^+^ M1 macrophages have also been observed in premorbid states, such as prediabetes, where these cells have been reported to share a direct correlation with HbA1C levels [130]. One of the most important markers for subclinical inflammation is C-Reactive Protein (CRP) [131], a pentameric acute-phase protein. Concerning macrophage modulation, CRP has been associated with M1 polarization mediated by stimulation of M-CSF secretion from endothelial cells and activation of NF-*κ*B [132, 133]. Finally, low adiponectin levels, as seen in the presence of visceral obesity, also favor M1 polarization, as this hormone is a promoter of M2 switching through AMPK, PPAR-*γ*, and PPAR-*α* [134]. Therefore, AMPK-activating drugs, such as metformin, may be useful in prevention of atherosclerosis [25, 26].

Other disorders featuring proinflammatory states, such as systemic lupus erythematosus (SLE) [135], rheumatoid arthritis (RA) [136], psoriasis [137], and antiphospholipid syndrome (APS) [138], have been associated with M1 polarization and higher risk for atherosclerosis and cardiovascular events. Reports on* in vitro* SLE models have described greater cholesterol accumulation in macrophages, along with increased IFN-*α* signaling and SR-A expression [135], as well as higher rates of macrophage-derived foam cell formation [139]. Likewise, subjects with SLE and RA exhibit higher levels of macrophage activation markers, such as neopterin [135] and macrophage migration inhibitory factor [140], as well as disorganized gene expression related to microbial translocation and immune dysregulation [141]. In APS, the TLR4 pathways appear particularly relevant as they intervene in the macrophage-foam cell transition induced by oxLDL/*β*2GPI/anti-*β*2GPI complexes [138] and participate in what has been denominated “autoimmune-mediated atherosclerosis,” seen in these individuals [142, 143].

On the other hand, M2 polarization requires attenuation of various M1-promoting intermediaries. In this regard, the p50 subunit of NF-*κ*B has been observed to inhibit NF-*κ*B-induced M1 polarization [144]. Similarly, complement protein C1q has been found to suppress NF-*κ*B activation in macrophages during lipoprotein endocytosis and processing, lowering release of inflammatory cytokines [145]. Other M2 promoters include Sphingosine-1-phosphate (S1P), an intermediary in the antiatherogenic functions of HDL-C, which increases IL-4 secretion and upregulates IL-4R*α* and IL-2R*γ* [146], and thioredoxin, which has been observed to potentiate IL-4-dependent M2-programming via downregulation of p16^INK4a^, with accompanying reduction of lesion area in animal models [147].

As can be observed, macrophage polarization within a certain scenario is a game of power, whose outcome is dictated by the prevailing kind of microenvironmental stimuli [148–150]. The complex molecular interplay underlying M1/M2 switching represents intriguing enigma, with important implications for therapeutic intervention in CVD.

## 6. Monocyte and Macrophage Migration

One of the earliest events in the pathogenesis of the atherosclerotic plaque is the adhesion of circulating monocytes to the vascular endothelium, followed by their migration to the intima. Various adhesion molecules (P-Selectin, ICAM-1, and VCAM-1), chemoattractant cytokines, and modified lipoproteins are responsible for monocyte recruitment to the intima [151], whereas* in vitro* models have shown lysophosphatidic acid and platelet-activating factor to be the main factors involved in their retention within the subendothelium [152]. In this location, monocytes differentiate into macrophages, able to capture oxLDL through scavenger-type receptors, and finally become foam cells, a cornerstone of atheromatous lesions [153], highlighting the importance of monocyte retention for plaque progression [154].

In this regard, emigration of monocyte-derived cells from atherosclerotic lesions has been associated with their regression; thus, decreased efflux of these cells may be a key phenomenon for their progression [152, 155]. Netrin-1, a protein associated with tumorigenesis and axonal migration, may play a major role in this scenario, by interfering with macrophage traffic in atherosclerotic lesions and perpetuating the local inflammatory response [156].

Lastly, macrophage proliferation is yet another fundamental finding in atherosclerosis: this mechanism may originate up to 90% of all locally accumulated macrophages [157]. Although the relative importance of these three pathophysiologic components, monocyte recruitment, macrophage retention, and macrophage proliferation, in atherosclerosis remains a provocative topic of debate, the former two phenomena appear to be the most dominant during the earlier phases of the disease, whereas macrophage proliferation may be particularly salient in its advanced stages [158]. At any rate, current experimental evidence suggests monocyte recruitment may be the most viable target for therapeutic modulation of the atherosclerotic process [159].

## 7. Macrophage Biomarkers: Role in the Inflammatory Response and Potential Clinical Applications

Inflammation is a major component of CVD and a fundamental driving force throughout all phases of atherosclerosis [160], with macrophages being the principal cellular mediators, by secreting a wide array of molecules, such as cytokines, chemokines, growth factors, and proteases, all of which are involved in different stages of the disease [161]. In this context, these products may be considered biomarkers, complying with the NIH definition [162].

Results from recent prospective studies have shown control of atherosclerosis may be poor, despite optimal risk factor management [163], and progression rates to be similar irrespective of age [164]. Indeed, although the accumulation of cellular functional alterations attributable to chronic disease and senescence is intimately linked to inflammation [165], entailing pathogenic mechanisms such as mitochondrial dysfunction, oxidative stress, and immune-endocrine aging [166, 167], the increasing prevalence of obesity, smoking, hypertension, and other risk factors in younger populations may be shifting this paradigm [17, 168, 169].

This alarming outlook accentuates the importance of assessing the potential benefits of targeting macrophage biomarkers for therapy, by either hindering proinflammatory pathways or enhancing anti-inflammatory mechanisms, while accounting for age and the impact of early intervention. Although many biomarkers have been associated with macrophage activation, their relationship with the different phenotypes remains unclear. Therefore, macrophage heterogeneity may represent a foundation for the study of their products' potential clinical applications, with respect to prediction of atherosclerotic progression and therapeutic modulation of the inflammatory response. This conundrum has already spawned a large body of research [170–172], Table 1. Nonetheless, to date, most of this research has been realized in animal models or* in vitro*, and many aspects remain incompletely elucidated in humans.

Interventions exploiting anti-inflammatory cytokines, such as IL-10, appear promising [173]. IL-10 has several antiatherogenic properties, regulating multiple immune functions and attenuating inflammation, chiefly through repression of IFN-*γ* [174]. Indeed, IL-10-deficient mice exposed to an atherogenic diet have been found to exhibit prominent IFN-*γ* expression, as well as greater-sized atherosclerotic lesions with significantly higher lipid deposition, lower collagen content, and increased T cell infiltration in comparison to mice with unaltered immune responses in equal conditions [175]. Similarly, Pinderski et al. [15] evidenced a significant decline in atherosclerotic progression in mice with T cells overexpressing IL-10, reflected in reduction of lesions, particularly in necrotic nuclei, and a pronounced decrease in lipidic accumulation in medium- and large-caliber arteries. Remarkably high IL-10 levels in specific areas within atherosclerotic lesions may obey a marked decrease in local expression of inducible Nitric Oxide Synthase (iNOS), along with macrophages with low apoptotic activity [176].

IL-13 has also demonstrated possibly exploitable antiatherogenic assets, as described by Cardilo-Reis et al. [16], who found IL-13 administration promoted favorable modifications in plaque morphology in LDL receptor-deficient mice, with increased collagen synthesis and decreased VCAM-1-dependent monocyte recruitment. Furthermore, a decline in macrophage concentration was observed in lesions, along with M2 polarization. In addition, mice with IL-13 deficiency developed larger-sized, more advanced plaques and appeared to synthesize lower levels of IL-4 and IL-10, disfavoring alternative activation of macrophages. Finally, IL-19 and IL-27 also boast notorious antiatherogenic properties: treatment with IL-19 has been associated with Th2 polarization of lymphocytes and decreased macrophage infiltration in atherosclerotic lesions in murine models [177]. Similarly, in animal models, administration of IL-27 has been described to diminish LDL-C traffic in macrophages, limiting generation of foam cells [18], and increase synthesis of ABCA1 via JAK2/STAT3 [178], restricting progression of atherosclerotic lesions.

Modulation of PPAR-*γ* in macrophages represents another alternative, due to their ability to inhibit expression of many inflammatory mediators [179]. Additionally, statin-mediated activation of PPAR-*γ* in macrophages has been linked with improved insulin sensitivity and M2 polarization, with reduced inflammation and atherosclerotic progression, expanding these agents' well-established therapeutic role in the management of CVD beyond the management of dyslipidemia [19, 180]. Indeed, statins boast a battery of pleiotropic effects, involved in anti-inflammatory, antioxidant, and immunomodulating mechanisms [181]. Youssef et al. [182] have proved statins to be able to shift the global immune environment towards anti-inflammation in murine models, with promotion of IL-4, IL-10, and TGF-*β* release and inhibition of proinflammatory messengers such as IL-12, TNF-*α*, and IFN-*γ*, powerfully inducing M2 polarization in murine models [20].

In light of these molecular findings, statins have been suggested for use in primary prevention, supported by research with animal [183–186] and human models [187, 188]. However, there remains a scarcity of evidence in certain specific groups of patients [189]; and reports associating these drugs with dysglycemia have also raised concerns regarding their possible widespread utilization in a primary preventive scenario [190, 191]. This relationship should be reassessed in studies using statin doses capable of influencing formation or stabilization of atherosclerotic plaques and levels of biomarkers such as CRP and TLR4 [21, 192–194].

Therefore, currently available evidence is insufficient to recommend statin therapy as a primary preventive strategy [195, 196]. Concerning secondary prevention, statins have been proven to be beneficial in patients with DM [197], hypertension [198], ST segment elevation myocardial infarction [199], and stroke [200]; and these effects may be present with both low-moderate and intensive statin therapy [201, 202]. Nevertheless, important side effects such as statin-associated myopathy [203–205] should be taken into account for long term pharmacological preventive interventions.

In any manner, statins may impact not only macrophages and endothelial cells, but also VSMC: pitavastatin and fluvastatin have been shown to lower expression of MCP-1 and TNF-*α* in these cells [206]. Likewise, atorvastatin has been observed to suppress TGF-*β*1-induced calcification of VSCM and promote autophagy via blunting of *β*-catenin signaling [207]. Similarly, other kinds of agents, such as telmisartan (an angiotensin II receptor antagonist), have been demonstrated to induce autophagy in VSMC via activation of PPAR-*γ* and AMPK and inhibition of mTOR [208].

**Figure 4:**
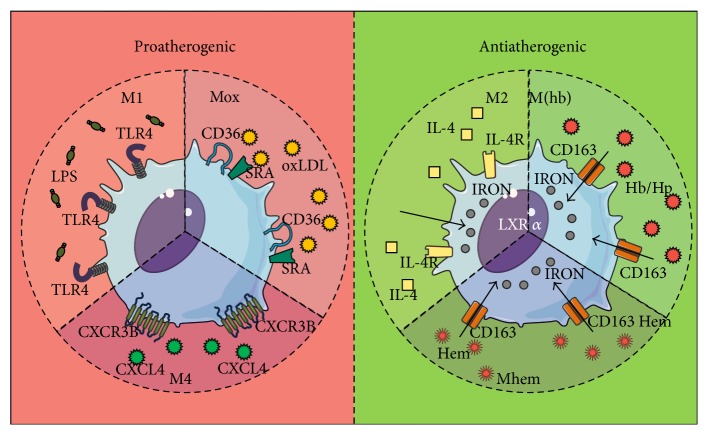
Proatherogenic and Antiatherogenic macrophages. Proatherogenic macrophages include M1 and Mox, whereas antiatherogenic macrophages include M2, M4, M(Hb), and Mhem. Each of these phenotypes varies in regard to associated stimuli and iron management.

Liver X Receptors (LXR) are important nuclear transcription factors in macrophages, with specific genomic targets, such as the LBP (Lipopolysaccharide Binding Protein) gene, involved in foam cell modulation via LXRa expression [22, 209, 210], and control of iron depots and iron-dependent oxidative stress [208]. Iron management profiles are especially important in macrophages, as they allow categorization of macrophages into pro- and antiatherogenic subtypes [211–214], Figure 4. Proatherogenic macrophage phenotypes include (a) M1 and (b) Mox, which exhibit preference for lipid oxidation and are Nfr2-modulated and located in active plaques [215]. Antiatherogenic phenotypes include (a) M2 and (b) M4, which are CXCL4-induced and express low CD163 levels [216], (c) M(Hb), which are activated by hemoglobin-haptoglobin complexes [217], and (d) Mhem, heme-inducible macrophages, which are characteristically located in intraplaque hemorrhage sites, with high heme oxidase levels induced by Activating Transcription Factor-1 (ATF-1) [23, 24, 213].

Other drugs, such as metformin, have also been described to intervene in macrophage functionality: this molecule can induce expression of AMPK and ATF-1, increasing heme concentration and thus driving Mhem polarization, ultimately preventing formation of foam cells [25]. Metformin has also been reported to lower ROS production and oxLDL-dependent cell death in human endothelial cells [26]. These pleotropic properties are considered to amplify this agent's effects on multiple cardiometabolic variables, such as lower mLDL-C levels [218] and enhanced activity of antioxidant enzymes like superoxide dismutase [219], and significantly attenuate the impact of atherogenic diets in vascular physiology [27].

In addition, microRNA (mRNA) molecules, short RNA sequences capable of regulating genetic expression, have recently claimed considerable interest as therapeutic targets in all genome-related diseases [220, 221], including atherosclerosis. These molecules may be exploited for the modulation of multiple inflammatory signaling cascades and macrophage polarization pathways [222]. Banerjee et al. [28] reported let-7c mRNA to suppress expression of M1 components in favor of a M2 phenotype, in consonance with similar descriptions by Zhuang et al. [223] in response to mRNA-233. Nevertheless, the study and application of mRNAs remain a vastly unknown yet encouraging field in regard to atherosclerosis and CVD.

Finally, regulation of NLRP3 inflammasome activation in M1 macrophages represents another novel alternative. In animal models, NLRP3 activation by ATP [224] and cholesterol crystals [225] is associated with increased lipid deposition and generation of foam cells, propelling its use as a marker for innate immune system activation in patients with coronary disease [226]. NLRP3 inhibition through silencing [29], or modulation with atorvastatin [31], ethanol [30], and scropolioside B [227], is associated with diminished oxLDL transport, NF-*κ*B inhibition, and deceleration of foam cell generation and plaque progression.

## 8. Concluding Remarks

The study of monocyte and macrophage heterogeneity has led to the identification of these cells' distinct functional phenotypes, which play dynamic roles in the onset and progression of atherosclerosis, as these cells may act as pro- or anti-inflammatory mediators, depending on the prevailing cues involved in their activation. A broad range of pre- and postpolarization biomarkers appear throughout this process, posing as valuable subjects for further investigation, in view of their possible applications in CVD.

As research efforts have advanced concerning macrophage activation and their associated biomarkers, new therapeutic targets have emerged in every stage of the atherosclerotic process. These innovative examples include modulation of macrophage autophagy [228–230], induction of IL-10 synthesis with conjugated linolenic acid [231], induction of M2 polarization through STAT3 via GLP-1 [232], inhibition of macrophage Notch1 [233], blocking of scavenger receptors such as CD68F [234], GABA- or topiramate-mediated inactivation of NF-*κ*B and MAPK^p38^ [235], and modulation of Treg cells in order to induce antiatherogenic macrophages [236]. Indeed, the future appears extraordinarily promising in this field, and further research is required in order to more thoroughly comprehend the molecular foundation of atherosclerosis and to identify possible interventional targets and their impact in the clinical setting.

## Figures and Tables

**Table 1:** Potential therapeutic targets in macrophage modulation.

Target/molecule	Study characteristics	Results	References
IL-10	Animal model: LDL receptor-deficient mice with IL-10 overexpression in T cells.	Monocyte/macrophage dysfunction, with reduced IFN-*γ* expression and foam cell apoptosis.	Pinderski et al. [15]

IL-13	Animal model: LDL receptor- and ApoE-knockout mice with atherosclerotic lesions that received exogenous IL-13.	LDLR−/− mice exhibited increased collagen synthesis and lower macrophage concentration in lesions. ApoE−/− mice showed decreased monocyte recruitment through VCAM-1, with possible alternative macrophage activation.	Cardilo-Reis et al. [16]

IL-19	Animal model: mice on atherogenic diets that received exogenous IL-19.	IL-19-treated mice showed lower amounts of macrophages, with Th2 polarization of circulating lymphocytes.	Johnson et al. [17]

IL-27	Animal model: mice deficient in IL-27 and IL-27 receptor.	Mice deficient in IL-27 and IL-27 receptor were more prone to atherosclerosis, with increased macrophage concentration in vascular walls, associated with higher oxLDL uptake and proinflammatory cytokine release.	Hirase et al. [18]

PPAR-*γ*	Animal model: mice without PPAR-*γ* in macrophages.	Mice without macrophagic PPAR-*γ* were more prone to obesity and insulin resistance; PPAR-*γ* activation appears necessary for alternative macrophage activation.	Odegaard et al. [19]

Statins	*In vitro* study: macrophages isolated from murine models were stimulated with IFN-*γ*, undergoing polarization towards M1 phenotype. These were exposed to varying doses of simvastatin in 9-hour cultures.	Exposure to simvastatin yielded an increase in IL-10 and CD206 expression and promoted M2 polarization.	Li et al. [20]
	*Ex vivo* study: carotid plaques from 140 patients were obtained, where 70 have preoperative statin use.	Those in the statin group showed lesser expression of TLR4 in macrophages and endothelial cells.	Katsargyris et al. [21]

LXR (Liver X Receptor)	Animal model: mice treated with LXR agonist T0901317.	Mice treated with T0901317 showed reduced macrophage infiltration and involution of plaques in early and late stages.	van der Stoep et al. [22]

Mhem induction	*In vitro* study: U937 cells (monocytic lineage) exposed to hemin (HO-1 activator) and IX Zinc Protoporphyrin.	Exposure to HO-1 inhibits expression of proinflammatory cytokines.	Ma et al. [23]
	*In vitro* study: macrophages from mice peritoneum and human atherosclerotic lesions.	The expression of HO-1 is a key in the acquisition of antioxidant activity in macrophages and is associated with decreased inflammation in the lesions.	Orozco et al. [24]

Metformin	*In vitro* study: endothelial cells from human coronary arteries, naïve and TRAF3IP2-deficient, exposed to HDL3, adiponectin, AICAR, and metformin.	Exposure to AMPK/Akt activators lowered production of superoxide, expression of TRAF3PI2, and oxLDL-C-mediated cell death.	Valente et al. [25]
	*In vitro *study: human monocyte-derived macrophages and mice bone marrow macrophages, exposed to heme and metformin.	Metformin induced activation of ATF1 at clinical concentrations (10 *μ*mol/L), with suppression of oxidative stress, enhanced cholesterol transport, prevention of foam cell formation, and suppression of macrophage activation.	Wan et al. [26]
	*Ex vivo* study: mononuclear cells from peripheral blood samples of healthy subjects, exposed to metformin, LPS, and Compound C (an AMPK inhibitor).	Monocyte-derived macrophages in the metformin group showed lower ROS production, with M2 polarization.	Bułdak et al. [27]

let-7c microRNA	*In vitro* study: M1 (GM-BMM) and M2 (M-BMM) macrophages derived from bone marrow of mice.	M2 macrophages showed higher let-7c mRNA levels. Their overexpression in M1 macrophages promoted shift towards M2 phenotype.	Banerjee et al. [28]

Modulation of NLRP3 inflammasomes	Animal model: ApoE knockout mice exposed to lentivirus.	Lentivirus silenced NLRP3, reducing plaque progression and local inflammation.	Zheng et al. [29]
	*In vitro *study: human macrophages treated with NLRP3 activators (ATP, cholesterol, serum amyloid A, and nigericin) and ethanol.	Ethanol attenuates macrophage activation and release of IL-1*β*.	Nurmi et al. [30]
	Clinical trial: 60 patients with coronary disease and 30 healthy subjects, randomly treated with atorvastatin or rosuvastatin. Expression of NLRP3 and IL-1*β* was assessed with reverse transcription-polymerase chain reaction.	Subjects treated with atorvastatin showed lower NLRP3 expression.	Satoh et al. [31]
